# The first serological survey of *C. Burnetii* in domestic animals in Poland

**DOI:** 10.1186/s12917-024-04423-y

**Published:** 2024-12-07

**Authors:** Hanna Turlewicz-Podbielska, Jakub Jędrzej Ruszkowski, Małgorzata Pomorska-Mól

**Affiliations:** 1 Department of Preclinical Sciences and Infectious Diseases, Faculty of Veterinary Medicine and Animals Sciences, Poznań University of Life Sciences, Wolynska 35, 60- 637 Poznań, Poland; 2 Department of Animal Anatomy, Faculty of Veterinary Medicine and Animals Sciences, Poznań University of Life Sciences, Wojska Polskiego 71C, 60-625 Poznań, Poland

**Keywords:** Dog, Cat, Rabbit, Q fever, Antibody

## Abstract

**Background:**

• Q fever, known as coxiellosis in animals, represents a global zoonotic threat caused by the intracellular bacterium *Coxiella burnetii* (*C. burnetii*). The disease affects many animal species, including those considered significant reservoirs, such as cattle, sheep and goats. Transmission of the pathogen to other domestic animals, including companion animals, and then to humans has also been observed, highlighting the importance of understanding the epidemiology and prevalence of *C. burnetii* among companion animals. The present study aimed to determine the frequency of seroreagents for *C. burnetii* within pet dogs, cats and rabbits from urban Poland areas and identify possible risk factors for these animals.

**Results:**

• In total, serum samples from 491 dogs, 427 cats and 93 rabbits were used in the study. The seroprevalence of anti-*C. burnetii* antibodies in dogs and cats reached 0.61% (3/491; 95% CI: 0.21–1.78) and 0.23% (1/427; 95% CI: 0.04–1.31), respectively. No significant differences in seroprevalence across species and different subpopulations (age group, gender, exhibited symptoms, or sampling location) were found. All 93 samples from rabbits were negative for anti-*C.burnetii* antibodies.

**Conclusions:**

• The seroprevalence rates of *C. burnetii* in dogs and cats were low; however, our results confirm that pet dogs and cats in Poland can be exposed to *C. burnetii* and may exhibit serological reactions. It has been reported that people who come into contact with secretions and excretions from the reproductive systems of dogs and cats (such as breeders, veterinarians, and veterinary clinic staff) may be at risk of contracting *C. burnetii*. Based on the findings, it is advised to be particularly cautious, especially when assisting with dogs and cats giving birth. Coxiellosis should be considered a potential cause of reproductive disorders in these animals. The results indicate that rabbits are probably less important in the circulation of the *C. burnetii* in the present study. This is the first serological survey of *C. burnetii* in pet dogs, cats and rabbits in Poland.

## Background

Q fever, also known as coxiellosis, is a widespread zoonotic infection caused by the intracellular bacterium *Coxiella burnetii* (*C. burnetii*). The pathogen affects various animal species, with cattle, sheep, and goats being the primary reservoirs [1]. Currently, the term “Q fever” is primarily linked to infections in humans, while “Coxiellosis” refers to the disease in animals [2, 3]. The bacteria are most heavily shed by animals through placentas and reproductive fluids, as well as in milk, vaginal secretions, faeces, and urine, leading to environmental contamination [4]. Pets can become infected through inhalation, consumption of infected placentas or milk, or via ticks [5]. However, in contrast to the majority of vector-borne diseases, ticks are not essential as vectors in the transmission of *C. burnetii* [1], and their role in the epidemiology of coxiellosis is being disputed due to the rare detection of *C. burnetii* in ticks. Also, *Coxiella*-like endosymbionts, which are non-infectious for the vertebrate hosts, have a strong genetic similarity to *C. burnetii*, and routine PCR detection usually cross-reacts, leading to misinterpretation of the real prevalence of *C. burnetii* in ticks [6, 7]. The main transmission route for domestic animals and humans is inhalation of contaminated aerosols [8]. Individuals with frequent exposure to *C. burnetii*-infected animals and their products, such as veterinarians, breeders, animal farmers, abattoir and tannery workers, and hunters, are more at risk of getting Q fever [3, 9]. Human infection can also result from consuming contaminated milk or dairy products [10]. In humans, the acute phase of Q fever presents as flu-like symptoms. In contrast, the chronic phase, more common among susceptible or immunocompromised individuals, can lead to severe pneumonia and endocarditis [4, 5]. In recent years, Europe has reported over 700 annual human cases of Q fever [11]. In Poland, analyses of Q fever outbreaks have identified two primary sources of human infection: the imported-infected animals and their products and domestic farm animals, primarily cows [9]. Infection with *C. burnetii* is a common problem in cattle herds in Poland. A seroprevalence study showed that 25.39% of Polish cattle tested between 2014 and 2017 had specific antibodies against *C. burnetii* [12]. In other research, the presence of the pathogen was confirmed by real-time PCR in 31.54% (88/279) of the tested herds [13]. Moreover, in the study conducted by Szymańska-Czerwińska et al., the average percentage of seropositive samples from Polish farm workers having contact with cattle and small ruminants during routine service (milking, veterinary service, or housekeeping tasks) was high and reached 39.07% (46/181) in ELISA [14]. According to the European Commission’s regulations, Q fever is classified as a category E disease, necessitating surveillance within the European Union. Additionally, under the Act on Animal Health Protection and Fighting Infectious Animal Diseases in Poland, Q fever is a notifiable disease [15]. Serological screening, mainly conducted using the ELISA tests recommended by the World Organization for Animal Health, is crucial for the monitoring of herd health status regarding zoonotic pathogens, including *C. burnetii* [16]. The role of pets, such as dogs, cats and rabbits, in *C. burnetii* transmission to humans is unknown, and serological studies in these species have not previously been carried out in Poland. Thus, the present study aimed to determine the frequency of seroreagents for *C. burnetii* within pet dogs, cats and rabbits from urban areas of Poland and identify possible infection risk factors for these animals.

## Methods

### Samples

**Fig. 1:**
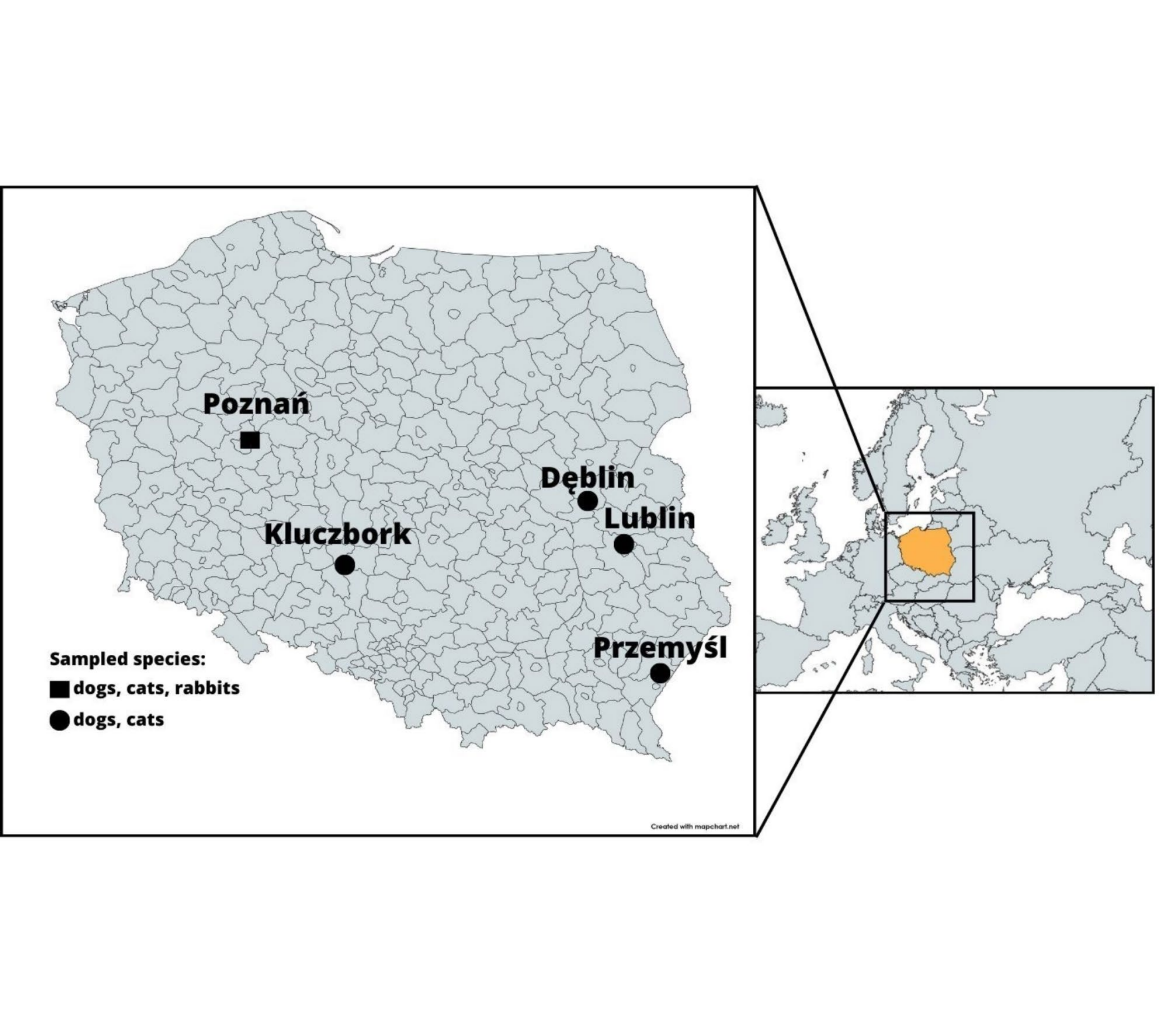
Map of sampling locations in Poland and animal species from which serum samples were collected. The map was generated using Mapchart https://www.mapchart.net and Canva https://www.canva.com

A total of 1,011 serum specimens gathered from October 2020 to July 2024 in six different veterinary practices across Poland (Poznań 52°24′24″N 16°55′47″E (wielkopolskie voivodeship); Przemyśl 49°47′05″N 22°46′02″E (podkarpackie voivodeship); Kluczbork 18°13′E 50°58′N (opolskie voivodeship); Lublin 22°34′E 51°15′N (lubelskie voivodeship); Dęblin 21°52′E 51°34′N (lubelskie voivodeship) (Fig. 1) were subjected to the present study. Samples from rabbits were obtained from a single practice (*n* = 93) and were sourced from animals in Poznań and the surrounding areas. The sera were preserved as previously reported by Turlewicz-Podbielska et al. [17, 18]. In total, the present study utilized serum samples from 491 dogs, 427 cats, and 93 rabbits. Pertinent information available for each sample included species, sex, age at sampling, location, and clinical presentation (healthy, presence of reproductive/respiratory/gastrointestinal/neurological/urinary disorders, or other symptoms).

### Detection of C. *burnetii* antibodies

An ELISA (ID Screen Q Fever Indirect Multispecies^®^, IDVet, Grabels, France) was used to detect anti-*C. burnetii* antibodies according to the manufacturer’s instructions. The kit’s manufacturer provided the diagnostic test’s sensitivity and specificity of 100%. The optical density (OD) was immediately measured at 450 nm in the Infinite^®^ 200 PRO microplate reader (TECAN, Mannedorf, Switzerland) after the reaction was stopped. The mean value of OD for the Positive Control (OD_PC_) and the mean value of OD for the Negative Control (OD_NC_) values were used to validate the test results. The results were interpreted using the cutoffs recommended by the manufacturer, based on the sample to positive ratio percentage (S/P%). This percentage was calculated using the following equation: S/P% = (OD_sample_ – OD_NC_)/(OD_PC_ – OD_NC_) x 100. Samples with S/P% ≥ 50% were deemed positive, those with S/P% ≤ 0.40 were classified as negative, and samples with S/P% ratio > 40 and < 50 were considered doubtful.

### Statistical analysis

The sampled animals were grouped based on their species into three categories. Then, subgroups were formed according to the available details at the time of sampling, such as gender, age group, location, and health status. RStudio version 4.1.2 was used for the analyses, except for the prevalence, which was calculated using an online program available at https://epitools.ausvet.com.au/ciproportion. The prevalence confidence intervals (CI) were calculated using the Wilson score method, whereas Pearson’s chi-square test was employed to analyze the data across different species, genders, health statuses, age groups, and locations. The odds ratio values were determined along with a 95% confidence interval to assess the risk of seroconversion in various animal species. A statistically significant p-value was considered to be less than 0.05.

## Results

**Fig. 2:**
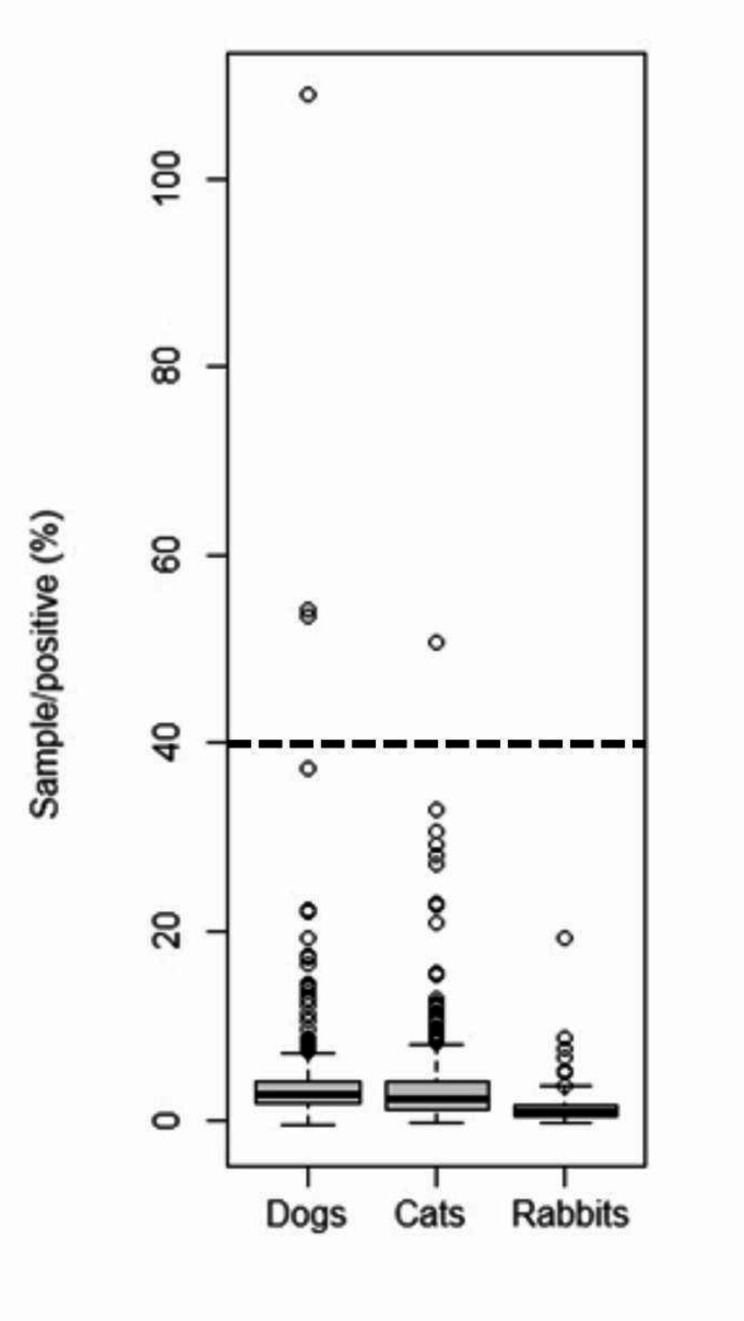
Boxplots of sample to positive (%) values obtained by ELISA in different species. A horizontal line indicates the cut-off point

The present study investigated the prevalence of anti-*C burnetii* antibodies in various pet animals. The serum samples were collected from dogs, cats, and rabbits. The overall seroprevalence of anti-*C. burnetii* antibodies were found to be 0.61% (3 out of 491) in dogs and 0.23% (1 out of 427) in cats, as detailed in Table 1. All rabbit serum samples tested negative for antibodies against *C. burnetii*. Statistical analysis did not reveal a significant correlation between the occurrence of antibodies and the animal species (*p* = 0.51). Further analysis involved calculating the odds ratios (OR) for the group with the higher seropositivity (dogs), with results presented in Table 2. When assessing the subsets of dogs, cats, and rabbits based on various criteria such as gender, age group, clinical symptoms, or sampling location, no significant differences in seroprevalence rates were observed. The mean OD in dogs was 0.093 (min. 0.037; max. 1.63), while the mean SP% was 3.83 (min. -0.49; max. 108.87). In cats, the mean OD was 0.087 (min. 0.04; max. 0.64, while the mean SP% was 3.83 (min. -0.41; max. 50.74). In rabbits, the mean OD was 0.06 (min. 0.04; max. 0.28, while the mean SP% was 3.83 (min. -0.34; max. 19.36). Figure 2 illustrates the serological prevalence in different species (sample to positive (SP%) values) using a boxplot.

**Table 1:** Seropositivity among cats and dogs split into risk factor groupings and detailed information about the structure of the sampled rabbit population

		Dogs				Cats				Rabbits
Variables		Total (positive)	Prevalence (%)	95% CI^a^	p-value	Total (positive)	Prevalence (%)	95% CI	p-value	No. of ind.^b^
Health status										
	Reproductive disorders	20 (0)			0.22	10 (0)			0.86	0
	Respiratory disorders	21 (0)				32 (0)				1
	Gastrointestinal disorders	85 (0)				46 (0)				6
	Neurological disorders	20 (1)	5.00	0.89–23.61		13 (0)				2
	Urinary disorders	21 (0)				80 (0)				2
	Cardiovascular disorders	16 (0)				11 (0)				0
	Others	150 (2)	1.33	0.37–4.73		134 (0)				25
	Healthy	153 (0)				101 (1)	0.99	0.17–5.40		57
	No data	5 (0)				0 (0)				0
Gender										
	Female	235 (0)			0.41	194 (1)	0.52	0.09–2.86	0.93	49
	Male	251 (3)	1.20	0.41–3.45		233 (0)				43
	No data	5 (0)				0 (0)				1
Age group (years)										
	< 1	63 (1)	1.59	0.28–8.46	0.09	46 (0)				17
	1–3	93 (2)	2.15	0.59–7.51		88 (1)	1.14	0.2–6.16	0.28	24
	4–7	99 (0)				98 (0)				33
	8+	231 (0)				195 (0)				10
	No data	5 (0)				0 (0)				9
Location										
	Poznań	371 (3)	0.81	0.28–2.35	0.91	330 (1)	0.30	0.05–1.7	0.99	93
	Lublin	48 (0)				62 (0)				0
	Przemyśl	15 (0)				15 (0)				0
	Dęblin	35 (0)				12 (0)				0
	Kluczbork	22 (0)				8 (0)				0
Total		491 (3)	0.61	0.21–1.78		427 (1)	0.23	0.04–1.31		93

^a^lower and upper values for the 95% confidence interval. ^b^No. of individuals

**Table 2:** Odds ratio calculated for dogs

Species	positive/total		OR^a^	95%CI^b^	*p*-value
Cats	1/427			Ref^c^	
Dogs	3/491		2.39	0.28–68.84	0.45

^a^OR: odds ratio. ^b^CI: lower and upper values for the 95% confidence interval. ^c^Ref: reference category

## Discussion

In the present study, no significant relationship was found between seropositivity and the health status of animals. The seropositive animals did not exhibit specific symptoms related to the reproductive system. According to medical records, one of the seropositive dogs was admitted with neurological symptoms, the second was diagnosed with babesiosis, and the third suffered from juvenile panosteitis. The seropositive cat did not show any symptoms of the disease. While rabbits can develop systemic *C. burnetii* infections, and females actively shed the bacterium into the environment through vaginal secretions [19], we did not find any seropositive rabbits in our study. It is essential to note that seropositivity signifies the presence of antibodies resulting from prior exposure to a pathogen; however, it does not confirm the presence of a specific antigen or indicate an active infection. The previous research findings indicate that dogs, cats, and rabbits can be infected with *C. burnetii* [20–25]. However, the pathogenesis of coxiellosis in domestic animals has not been well-defined yet, and is probably similar to that established for other mammals [24–26]. The pathogen enters the body through the mouth and nose and spreads to various organs, with a preference for the reproductive system, particularly the uterus and mammary glands in females [21]. Consequently, pregnant animals often experience significant clinical symptoms, such as abortions, stillbirths, the birth of small or weak offspring, and mastitis [1, 3]. Reproductive abnormalities, such as dystocia, stillbirths, and perinatal mortality, have been observed in dogs and cats due to *C. burnetii* infection [20, 24]. Many bacteria are excreted during delivery, similar to ruminants. Clinically healthy parturient queens shed a considerable number (10^9^ per g of tissue) of bacteria into the environment during parturition [21]. Owners should be observant of reproductive abnormalities in their pets and seek specific laboratory tests to begin treatment and preventive measures promptly if an animal is found to be infected. Other non-specific symptoms may also be evident in infected animals. In cats, experimental infection with *C. burnetii* can cause fever, loss of appetite, and lethargy [22]. In laboratory animals, *C. burnetii* inoculation in guinea pigs and mice results in pneumonia, hepatitis, and splenomegaly [27, 28]. In cattle, inflammation of cardiac valves and chronic endometritis leading to chronic subfertility have been reported [29]. However, it is important to note that *C. burnetii* infections in animals often occur without visible symptoms [24], and coxiellosis in dogs and cats is frequently undiagnosed [30]. Coxiellosis should be considered in the differential diagnosis, especially when medical history indicates possible contact with *C. burnetii*.

We have noted very low seroprevalence of *C. burnetii* among dogs and cats in our study (0.61% and 0.23%, respectively). The data regarding seroprevalence in pet dogs, cats and rabbits in European countries is scarce. Low seroprevalence in pet dogs was observed in 1992 in Bologna, Italy, where it reached 0.9% (7/802) [31] and in Montenegro in 2019 − 1.2% (3/259) [32]. Our results are also similar to those observed In Portugal, where a decrease in the exposure to *C. burnetii* was observed in cats from 17.2% (5/29) in 2012 to 0.0% (0/47) in 2021 and in dogs from 12.6% (19/151) in 2012 to 1.7% (1/60) in 2021 [30]. In different studies, the observed seroprevalences in dogs and cats were higher. In dogs from Southeastern France, it was 31.2% (165/528) [33]. In southern Italy, it reached 5.97% (16/268) in dogs using a commercial multispecies ELISA [34]. The seroprevalence in free-roaming cats in Spain was also higher than in our study (37%; 108/291) [35]. Meredith et al. observed extremely high seroprevalence in cats in the United Kingdom (61.5%; 16/26); however, a small number of samples were examined in this study [36]. The highest seroprevalence in our study was observed in dogs, and we have found one seropositive cat. However, we did not observe an association between seropositivity and species. In general, cats are thought to be predisposed to exposure to *C. burnetii*, possibly due to their living habits. Domestic cats frequently have unrestricted access to the outdoors, allowing them to engage their hunting instincts, and potentially come into contact with infected wildlife prey [26]. We found that the prevalence of *C. burnetii* in cats was lower than in dogs. The cats in our study came from urban areas where most of them are kept indoors, with limited access to the outside environment. On the other hand, dogs are taken for walks daily and have potential contact with wild reservoirs of coxiellosis, such as wild rodents, ungulates, and birds [36, 37]. The etiological agent of coxiellosis may also come from pet food. Recent years have shown an increasing trend in using less traditional (alternative) feed ingredients in pet foods, especially in dogs fed with raw meat or with allergies or food intolerances. It is worth mentioning that recently (June 2023), a fatal disease outbreak in cats associated with poultry meat occurred in Poland. Most cases tested in the country (29/47) were positive for the highly pathogenic avian influenza (HPAI) A (H5N1) virus. Cat viral sequences were highly similar, belonging to clade 2.3.4.4b and cluster with virus strains from birds sampled in Central Europe from late 2022 onwards. These findings suggest a potential common infection source and food samples from affected households were tested for the HPAI H5N1 virus. The pathogen was detected in one poultry meat sample [38]. Raw meat diets containing reservoir species may provide a source of *C. burnetii* transmission. The presence of *C. burnetii* DNA in raw meat packages for pet consumption has been demonstrated previously. Shapiro et al. [39] found the pathogen in kangaroo meat packages. Due to the limited number of studies, the prevalence of *C. burnetii* infection among rabbits in Europe remains to be determined. To date, there have been no studies investigating the presence of antibodies against *C. burnetii* in pet rabbits. Although pet rabbits typically do not encounter potential reservoirs of this disease or environments that may be contaminated, the possibility of infection cannot be entirely excluded. For example, such an occurrence could arise from exposure to contaminated raw vegetables or fresh plants sourced from unknown origins. Studies conducted so far are focused on wild lagomorph populations of Spanish Mediterranean ecosystems, and the seroprevalences among these populations are higher than in our study due to contact with wild and domestic ungulates [40]. Recently, Castro-Scholten et al. reported that 11.3% (53/471) of European wild rabbits had antibodies against *C. burnetii* in their study [40]. Higher seroprevalence (65.5%; 394/602) was also found in wild rabbits from central areas of this country [25]. The relatively low occurrence of antibodies in pet dogs and cats and the absence of any signs of infection in pet rabbits in our study could be attributed to our sampling of animals from urban areas. In these areas, most animals are kept indoors, have access to high-quality veterinary care, and pets likely have minimal contact with the pathogen. Similarities and differences between the studies cited resulted from the type of population, the year, and the epidemiological scenario of the country taken into consideration.

Our results indicate no significant relation between seropositivity and species, age group, gender or sampling location. In the present study, all seropositive animals were younger than 3 years. All seropositive dogs were males, and one seropositive cat was female. All the seropositive animals came from Poznań city. A lack of association between gender and seropositivity in dogs and cats was observed previously in other studies [26, 30]. On the other hand, female dogs were statistically more seropositive than male dogs in the study by de Franca et al. [41]. In 2022, Anastácio et al. [30] reported that the proportion of positive results was slightly higher in female cats, which is consistent with our study. Shapiro et al. also found more seropositive female cats than males (5/300 and 18/300, respectively) [42]. Our results regarding age are consistent with those obtained by Shapiro et al. [42]. They found most of the seropositive cats in the age group 1-2.5 years. Conversely, Anastácio et al. observed a higher proportion of positive results in dogs and cats older than 24 months [30], and Ebani et al. found most of the seropositive cats in animals older than 5 years [26]. These differences may result in different population structures around the world.

Pet animals, especially those in close contact with their owners, have been suspected of acting as reservoirs of *C. burnetii* during urban Q fever/coxiellosis outbreaks [24]. Globally, dogs and cats play a minor role in transmitting *C. burnetii*. However, infected pets may pose a severe public health threat. A few outbreaks related to pet transmission have been reported, in which affected individuals confirmed the contact with parturient animals and tested positive for *C. burnetii*. An outbreak of Q fever occurred among the staff members of a small animal veterinary hospital in Sidney, Australia [43]. Nine veterinary personnel tested positive for Q fever based on serological and/or PCR tests. Eight of the affected persons had worked on the day a cesarean section was performed on a queen, while the ninth person handled the equipment used during the cesarean section the following morning [43]. In 2018, Malo et al. documented a human outbreak of Q fever in southeast Queensland, Australia [44]. The outbreak involved two individuals working in a veterinary clinic and four workers at an animal refuge who developed diseases after being exposed to a parturient queen cat and her litter, which were euthanized on the same day as the birthing event. Laboratory diagnostics using serological and molecular methods confirmed *C. burnetii* infection in the patients. Additionally, a single human case report has been linked to a dog infection during a Q fever outbreak, where three family members developed pneumonia twelve days after being exposed to an infected whelping dog [20]. Regrettably, the entire litter of the infected dog did not survive [20]. Our study confirmed the exposure to *C. burnetii* in both cats and dogs. From the One Health approach to coxiellosis in animals, this study highly recommends following proper hygiene and veterinary measures, especially in people with constant contact with dogs and cats, since vaccines to prevent coxiellosis in these species are unavailable. To minimize the risk of infection in pets, treatment against hematophagous arthropods is pivotal. Furthermore, dogs and cats should be kept far from food originating from *C. burnetii*-infected animals (raw milk, urines, faeces, genital secretions, placentae, and aborted fetuses). Control measures against commensal rodents by maintaining good hygiene conditions and using traps help reduce occasions of predation. People exposed to contact with dogs and cats, especially secretions and excretions of the reproductive system (breeders, veterinarians and other personnel of veterinary clinics), must exercise caution, including wearing masks, goggles, and gloves, when attending to dogs or cats during abortion or delivery and in the subsequent days. For example, being a veterinarian or veterinary student was significantly associated with *C. burnetii* seropositivity (prevalence odds ratio equal to 6.1) compared to participants who did not work in contact with animals [45]. Furthermore, it is imperative to implement measures aimed at preventing and mitigating environmental contamination after abortion or parturition events. It is essential to properly dispose of placentae, fetuses, and materials contaminated in the process. Parallel to this, a rigorous protocol for the disinfection of environments and instruments engaged in these procedures must be established and adhered to to safeguard against the propagation of contaminants [46].

Our study has some limitations. It must be underscored that serological assessments may not serve as a reliable indicator for ascertaining the role of specific animals as potential vectors for *C. burnetii* transmission to humans. The seropositivity observed in both canines and felines for *C. burnetii* merely reflects previous exposure, offering no definitive information regarding the timing of exposure. It does not clarify whether the exposure culminated in either a clinical or a subclinical manifestation of the disease. ELISA and Indirect immunofluorescence assay (indirect IFA) are preferred as rapid screening tools for coxiellosis. IFA is suggested to be more accurate for determining the serological status of individuals and is currently recommended as a gold standard in the diagnosis of Q fever in humans [14]. The indirect IFA can distinguish between acute and chronic infections. However, commercial kits are not available for veterinary use [47]. Further studies comparing results obtained by ELISA and IFA are recommended. Moreover, elucidation of the risk factors contributing to the intricate transmission dynamics of *C. burnetii* among domestic animals will substantially augment our comprehension of the epidemiological patterns of Q fever/coxiellosis. Given the notable zoonotic capabilities of *C. burnetii*, a deepened understanding of its transmission dynamics within pet populations is vital. Such knowledge will significantly aid in formulating robust public health strategies and establishing vigilant surveillance frameworks to curtail the human infection risk posed by this disease.

## Data Availability

The data that support the findings of this study are available from the corresponding author upon reasonable request. Data are located in controlled access data storage at Poznan University of Life Sciences.

## Abbreviations

C. burnetii: Coxiella burnetii
DNA: Deoxyribonucleic acid
ELISA: Enzyme-linked immunosorbent assay
HPAI: Highly pathogenic avian influenza
H5N1: Type 5 hemagglutinin protein and a type-1 neuraminidase protein
IF: Immunofluorescence assay
OD: Optical density
OR: Odds ratio
SP: Sample to positive values
